# When Pain Cannot Protect: Self-Harm Risk in Congenital Insensitivity to Pain With Emotionally Unstable Personality Disorder

**DOI:** 10.1192/bjo.2026.11884

**Published:** 2026-06-30

**Authors:** Gayathri Rangith

**Affiliations:** Cygnet Health Care, Oldbury, United Kingdom

## Abstract

**Aims:**

Congenital insensitivity to pain (CIP) is a rare neurogenetic condition characterised by lifelong absence of nociception, removing a fundamental biological deterrent to injury. Psychiatric risk-assessment frameworks implicitly rely on pain perception, distress cues, and behavioural feedback to signal escalation. We describe a case of severe self-harm escalation in a young adult with genetically confirmed CIP, emotionally unstable personality disorder (EUPD) highlighting critical limitations of standard inpatient risk-management approaches and the need for pain-independent safeguarding strategies.

**Methods:**

We describe a female in her early twenties admitted to an acute psychiatric ward under the Mental Health Act following overdose and repeated self-harm. She has a childhood diagnosis of genetically confirmed congenital insensitivity to pain due to a homozygous SCN9A mutation, with lifelong absence of pain perception and a history of significant injuries without reported pain. She also has longstanding neurodevelopmental and personality vulnerabilities, including autism spectrum traits and emotionally unstable personality disorder, with self-harm beginning in early life. During admission, she described persistent internal voices and intrusive phenomena consistent with pseudo-hallucinations associated with EUPD and autism, rather than primary psychotic illness. Despite prior minor self-harm episodes, environmental access to hazards such as sharps and boiling water continued. This culminated in a severe self-inflicted scald injury requiring admission to a specialist burns unit and intravenous antibiotics, with minimal behavioural or verbal pain response observed during injury assessment and wound care.

**Results:**

This case demonstrates how self-harm can escalate rapidly and silently when nociceptive feedback is absent. The absence of pain and distress cues rendered conventional observation levels and harm-minimisation strategies ineffective, delaying recognition of injury severity. Risk could not be reliably inferred from reported experience, expressed intent, or diagnostic category. Even in the absence of primary psychosis, self-harm associated with EUPD and autism progressed to life-threatening physical injury without typical warning signals, exposing a critical blind spot in inpatient psychiatric risk frameworks that rely on pain-dependent deterrence and behavioural escalation markers.

**Conclusion:**

In patients with congenital insensitivity to pain, self-harm risk must be assessed independently of pain perception and expressed distress. Psychiatry services should implement pain-independent safeguards, including early and sustained environmental restriction, routine body mapping, scheduled wound and physical observations, and prioritisation of psychotic symptom control. This case highlights the need for integrated neuropsychiatric approaches and revised inpatient risk frameworks when biological pain deterrents are absent.

